# Antibody Based Delivery of Toxins and Other Active Molecules for Cancer Therapy

**DOI:** 10.3390/biomedicines10020267

**Published:** 2022-01-26

**Authors:** Letizia Polito

**Affiliations:** Department of Experimental, Diagnostic and Specialty Medicine—DIMES, Alma Mater Studiorum—University of Bologna, Via S. Giacomo 14, 40126 Bologna, Italy; letizia.polito@unibo.it

The use of radio- and chemotherapeutic agents in cancer therapy have demonstrated evident antitumor effects, but also limitations (remarkable side-effects due to lack of selectivity for tumor cells, development of drug resistance, and occurrence of secondary malignancies). As a consequence, the study and development of alternative therapies, such as immunotherapy, has been deeply stimulated, in order to find therapies with greater specificity for transformed cells and less nonspecific toxicity. The advantage of immunotherapy lies in its characteristics (the recognition of specific targets on the cell membrane), which are completely independent from the parameters on which chemotherapy and radiotherapy are based. This results in a lack of superimposition of side-effects and unimpaired cytotoxicity, towards cell clones resistant to chemotherapy and radiotherapy. Today, inspired by Ehrlich’s “magic bullet” concept, one of the most promising research approaches is based on the linking of pharmacologically active molecules to carriers, mainly antibodies, for selective delivery to target cells. These hybrid conjugates are primarily applied to research in the field of cancer therapy [1].

Thus, most immunotherapeutic approaches are focused on the targeting of specific antigens on the surface of cancer cells. An essential requirement for this approach is that the target molecule is confined to the cell population to be destroyed, or at least that the target molecule is not present on stem cells or other cell types that are essential for organism survival. Antibodies are the most utilized carriers, due to their stability in blood and avidity and affinity for the target antigen. Many different molecules have been exploited as toxic moieties; the most studied are toxins (bacterial or plant), drugs, radionuclides, and human enzymes. The most used bacterial toxins are Pseudomonas Exotoxin A (PE) [2] and the Diphtheria toxin (DT) [3], which inhibit translation through the NAD-dependent ADP-ribosylation of the elongation factor-2, causing cell death. The most commonly used plant toxins for therapeutic purposes are ribosome-inactivating proteins (RIPs) [4,5], mainly ricin [6] and saporin [7]. RIPs are also known as polynucleotide: adenosine glycosylases [8] because they are able to remove adenines from many different polynucleotide substrates, causing cell death through several mechanisms [9,10,11].

The collection of five scientific articles composing this special issue highlights the progress in the knowledge of the antibody-based delivery of toxins and other active molecules against malignant cells, thus underlying their potential in anticancer therapy.

As mentioned above, the identification/selection of valid targets is a strategically important action to approach the immunotherapy of a specific cancer. Nectin cell adhesion molecule 4 (NECTIN4) is a potential therapeutic target for cutaneous squamous cell carcinoma, the second most common skin cancer. The expression of NECTIN4 was found in most of cutaneous squamous cell carcinoma-studied tissues and on the plasma membrane of the A431 cell line. NECTIN4 was demonstrated to have a role in the regulation of cell–cell interactions, in the migration and proliferation of cutaneous squamous cell carcinoma cells [12]. Prostate-specific membrane antigen (PSMA) represents a reliable marker, ideal for imaging and therapy in prostate cancer (PCa). The validity of the anti-PSMA antibody fragment scFvD2B as a theranostic tool was exploited in a review article combining the characterization of its biomolecular and tissue cross-reactivity characteristics with a comprehensive summary of what has already been performed in preclinical models, to evaluate imaging and therapeutic activities. The authors concluded that scFvD2B being a versatile and robust molecule, with sufficient tumor-to-background ratios for targeting and imaging PSMA-expressing cancer, can be considered a good potential reagent for PCa diagnosis and treatment [13].

Two review articles exploited the possibility of application of immunoconjugates (IC) for the therapy of colorectal cancer [14] and sarcoma [15]. 

Colorectal cancer (CRC) is the second most lethal cancer. Several monoclonal antibodies (mAbs) are included among the treatment options for advanced CRC, but often they may have limited clinical activity. The review article by Sanz et al. [14], reported data about the wide variety of ICs tested in preclinical studies and clinical trials for CRC treatment. For each conjugate, the target, the carrier and payload are described in detail and the preclinical efficacies in vitro and in vivo are reported. To date, seven conjugates have entered CRC clinical trials, two containing the plant toxin ricin and five containing the bacterial toxin PE. Moreover, the challenges and future directions in immunotoxin (IT) design were analyzed. 

Sarcomas are a heterogeneous-low-incidence group of malignancies that arise from mesenchymal tissue. Rare in adults, sarcomas are more frequent among pediatric tumors. The main therapy for sarcoma treatment is surgery, accompanied by neoadjuvant or adjuvant chemotherapy/radiotherapy. In this special issue, the review article by Polito et al. [15] reported preclinical and clinical data, regarding different type of ICs, antibody–drug conjugates, immunotoxins and radioimmunoconjugates. Many details are given about the choice of the target, the carrier and the payload; the results obtained in vitro, in animal models and in patients are reported and discussed. Currently, seven antibody–drug conjugates and two radioimmunoconjugates are under phase 1–2 clinical trials for sarcoma therapy, and a large number of ICs have been evaluated in preclinical studies. The results obtained with ICs in these preclinical sarcoma models encourage the translation to further clinical trials.

The clinical development of ITs has been hampered by some side effects, mainly represented by their immunogenicity and the development of dose-limiting toxicities, which can derive from nonspecific absorption by non-target cells. An important step forward in the clinical development of ITs could be given by widening the therapeutic window of the targeted toxins, in order to obtain a useful therapeutic effect at a lower dose. The cytotoxicity of the RIP saporin, and of saporin-containing ITs, has been shown to augment when given to cells with the triterpenoid saponins at sub-toxic concentrations. This is probably due to the endolysosomal escape of the toxin to the cytosol. Several inhibitors of endocytic processes were used to investigate the role played by saponin in the endolysosomal escape of saporin and a saporin-containing IT targeting CD38. The inhibitors of clathrin-mediated endocytosis, micropinocytosis, and endosomal acidification annulled the saponin-induced increase in the endolysosomal escape of the toxin into the cytosol. A comparison of the effect of the inhibitors, and the correlation between toxin augmentation and escape, indicates that increasing endolysosomal escape of the toxin is at least one of the contributory mechanisms by which saponin augments saporin cytotoxicity [16].

In conclusion, the studies collected in this special issue confirmed the potential of immunotherapy for targeted therapy on different cancer models. It is possible to believe that, in the near future, antibody-based therapeutic approaches could improve the outcomes of cancer patients, overcoming some difficulties associated with standard therapy.
